# Applications of Herbal Medicine to Treat Autosomal Dominant Polycystic Kidney Disease

**DOI:** 10.3389/fphar.2021.629848

**Published:** 2021-04-27

**Authors:** Guangying Shao, Shuai Zhu, Baoxue Yang

**Affiliations:** ^1^ State Key Laboratory of Natural and Biomimetic Drugs, Department of Pharmacology, School of Basic Medical Sciences, Peking University, Beijing, China; ^2^ Key Laboratory of Molecular Cardiovascular Sciences, Ministry of Education, Beijing, China

**Keywords:** chronic kidney disease (CKD), autosomal dominant polycystic kidney disease (ADPKD), herbal medicine, therapy, pathogenesis

## Abstract

Autosomal dominant polycystic kidney disease (ADPKD) is a common hereditary kidney disease, which is featured by progressively enlarged bilateral fluid-filled cysts. Enlarging cysts destroy the structure of nephrons, ultimately resulting in the loss of renal function. Eventually, ADPKD develops into end-stage renal disease (ESRD). Currently, there is no effective drug therapy that can be safely used clinically. Patients progressed into ESRD usually require hemodialysis and kidney transplant, which is a heavy burden on both patients and society. Therefore, looking for effective therapeutic drugs is important for treating ADPKD. In previous studies, herbal medicines showed their great effects in multiple diseases, such as cancer, diabetes and mental disorders, which also might play a role in ADPKD treatment. Currently, several studies have reported that the compounds from herbal medicines, such as triptolide, curcumin, ginkolide B, steviol, *G. lucidum* triterpenoids, Celastrol, saikosaponin-d, *Sparganum stoloniferum* Buch.-Ham and Cordyceps sinensis, contribute to the inhibition of the development of renal cysts and the progression of ADPKD, which function by similar or different mechanisms. These studies suggest that herbal medicines could be a promising type of drugs and can provide new inspiration for clinical therapeutic strategy for ADPKD. This review summarizes the pharmacological effects of the herbal medicines on ADPKD progression and their underlying mechanisms in both *in vivo* and *in vitro* ADPKD models.

## Introduction

Autosomal dominant polycystic kidney disease (ADPKD) is the most common hereditary kidney disease, with the current incidence of 1/1,000–1/400 worldwide. The course of the disease is characterized by multiple growing cysts in bilateral kidneys. The cysts are derived mainly from renal tubular epithelium. As the cyst getting larger, the cysts compress the normal renal parenchyma, thus resulting in the destruction of the normal renal structure and function (Bergmann et al., 2018).

*Pkd1* and *Pkd2* are two main genes participating in the pathogenesis of ADPKD, which encode protein polycystin 1 (PC1) and polycystin 2 (PC2), respectively (Cornec-Le Gall et al., 2019). *Pkd1* gene mutation accounts for approximately 85–90% of total ADPKD patients, and 10–15% ADPKD patients are caused by *Pkd2* gene mutation. PC1 localizes to the primary cilia, plasma membrane and adhesion complex in polarized epithelial cells. PC2 is a six-transmembrane protein and functions as a Ca^2+^-responsive cation channel of the transient receptor potential family, and co-localizes with PC1 to the cilium and plasma membrane. Proteins PC1 and PC2 work together to form the PC1-PC2 complex, which is activated in reaction to ciliary bending and then leads to the release of Ca^2+^ from the intracellular store, thus inducing signal transduction. The disruption of the functional PC1/PC2 complex abolishes normal cellular Ca^2+^ signaling, which subsequently increases intracellular cAMP and activates PKA signaling pathway, then activating downstream proliferative signalings (Yamaguchi et al., 2006). During the progression of ADPKD, some signaling pathways are activated, which are related to hyperproliferation, fluid secretion and fibrosis, such as mTOR, JAK, Wnt and MAPK signaling pathways. And some other pathways are suppressed, for example, AMPK pathway, which participates in intracellular energy generation.

However, existing drugs have their own limitations in ADPKD therapy. At present, tolvaptan, the first FDA-approved drug, is available clinically for ADPKD patients. Tolvaptan acts as a V2R antagonist, and functions by downregulating intracellular cAMP level to inhibit the abnormal proliferation in ADPKD kidneys. However, a signal of liver toxicity risk emerged with tolvaptan application, which is marked by extremely elevated ALT level and needs to be carefully monitored during the therapy. Another drug of vaptans, Lixivaptan, which is a newer, nonpeptide, oral V2R-specific antagonist, exhibits relatively preserved renal function (stage 1 and 2) and moderately impaired renal function (stage 3). Another family of drugs are the analogs of somatostatin, lanreotide and octreotide, which slow the progression of ADPKD by inhibiting the chlorine channel. However, they did not show significant effect on the renal function in clinical trials.

In addition, interventions to the abnormal signaling pathways, metabolic and dietetic approach are also regarded as possible strategies for ADPKD drug development. For example, mTOR inhibitors (everolimus and sirolimus), metformin (an agonist of AMPK), 2-deoxy glucose (2DG) (a glucose analog that can paralyze the glycolytic pathway), tyrosine kinase inhibitors (bosutinib and tesevatinib) and caloric restriction diet are all reported to retard cyst growth in ADPKD patients to different extent (Testa and Magistroni, 2020). But none of these drugs mentioned above is considered as satisfactory therapeutic agents for ADPKD for their drawbacks, such as unstable effects on slowing the progression of the disease, restricted applications of the aquaretic type drugs and little effect on the loss of renal function. Therefore, there still remains an urgent demand for alternative effective drugs.

**FIGURE 1:**
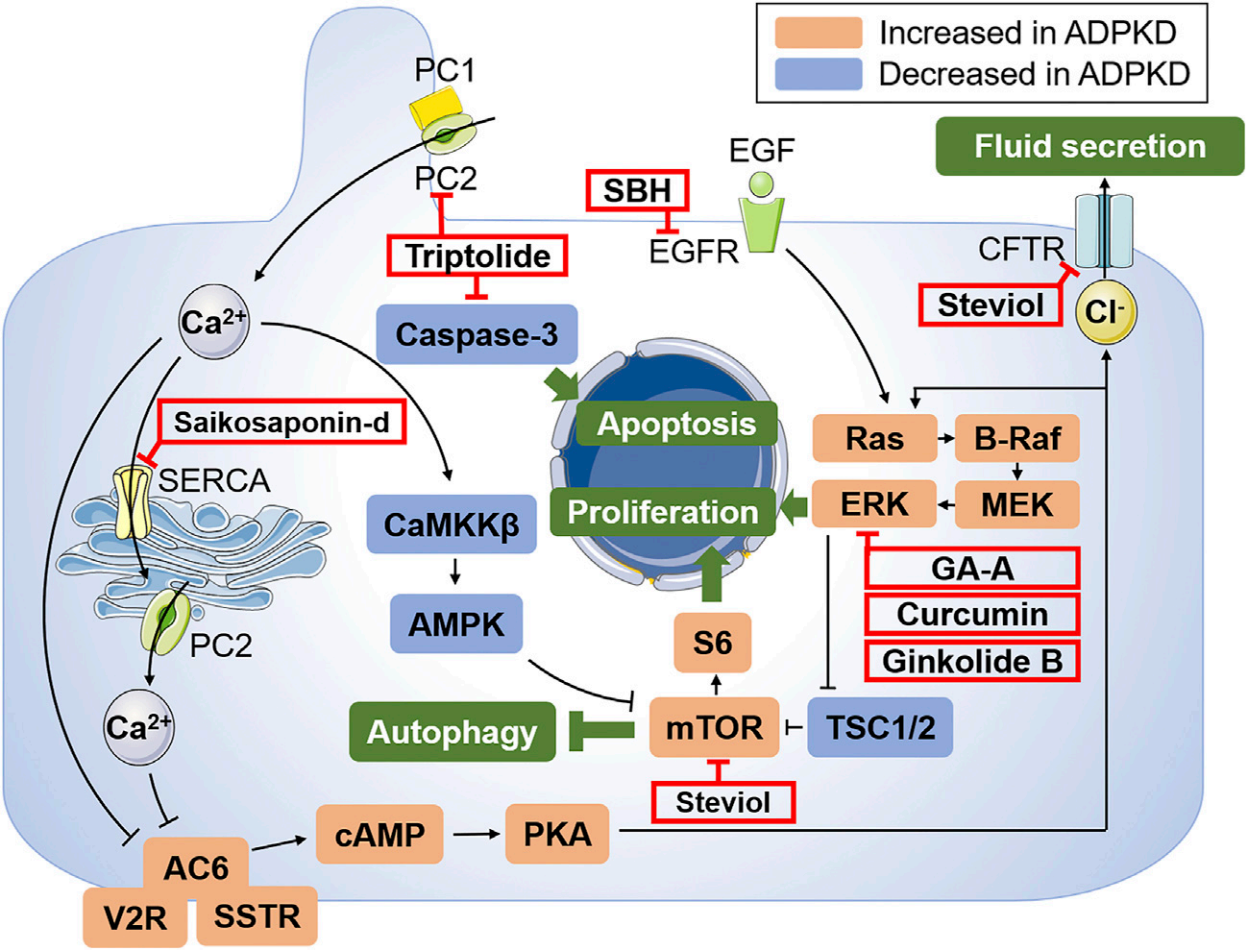
Schematic illustration of the key mechanisms of ADPKD pathogenesis and targets of potential treatments of herbal medicines. PC1 and PC2 express in different subcellular locations and aberrant function of PC1 and PC2 results to decreased intracellular Ca^2+^ and subsequent activation of cAMP *via* AC6. Abnormally increased cAMP leads to the activation of PKA, activating Ras/B-Raf/MEK/ERK and mTOR signaling pathways through inhibiting TSC1 and TSC2, which drive cell proliferation. Moreover, the activated PKA can also promote the transport of Cl^−^ into the cyst cavity *via* CFTR, thus causing increased cyst fluid secretion. Autophagy can also be induced by Ca^2+^-mobilizing agents by activating the CaMKKβ-AMPK-mTOR signaling cascades. In addition, EGF activates Ras and promotes cell proliferation by binding to the corresponding receptor. The targets of candidate drugs are depicted as red boxes. Abbreviations: PC1, polycystin-1; PC2, polycystin-2; AC6, adenylyl cyclase six; V2R, vasopressin type 2 receptor; SSTR, somatostatin receptor; cAMP, cyclic adenosine monophosphate; PKA, protein kinase A; CFTR, cystic fibrosis transmembrane conductance regulator; EGF, epidermal growth factor; EGFR, epidermal growth factor receptor; MEK, mitogen activated protein kinase; ERK, extracellular-signal regulated kinase; TSC, tuberous sclerosis; mTOR, the mammalian target of rapamycin; SERCA, sarcoplasmic/endoplasmic reticulum Ca^2+^ atpase; CaMKKβ, Ca^2+^/CaM-dependent protein kinase β; AMPK, AMP-activated protein kinase; GA-A, ganoderic acid A; SBH, *Sparganum stoloniferum* Buch.-Ham.

The therapeutic properties of plants have long been recognized to treat various diseases for centuries. At present, a number of plant natural products have either been clinically used to treat a variety of human diseases, or have shown particularly interesting biological activities that are worth further exploration (Li and Weng, 2017), such as artemisinin from *artemisia annua*, which has great anti-malaria effect and won the Nobel prize in 2015. Recently, some studies have found that herbal medicines play different pharmacological roles in renal diseases including ADPKD (Wang et al., 2019). Several natural herbal medicines have been reported to restrain cystogenesis and improve renal function in animal models of ADPKD (Yuajit and Chatsudthipong, 2016). In this article, we summarized the pharmacological effects of several herbal medicines on ADPKD and the underlying mechanisms (Figure 1), expecting to provide new prospectives for ADPKD treatment.

## Pharmalogical Effect of Herbal Medicine on ADPKD

### Triptolide

Triptolide is a diterpene triepoxide that was first isolated from the medicinal plant *Tripterygium wilfordii* Hook F (TWHF) and its structure was characterized in 1972. Its molecular formula is C_20_H_24_O_6_ and the molecular mass is 360 (Wei et al., 2019). Clinical and experimental studies have shown that triptolide has anti-inflammatory and immunosuppressive activities in bone marrow, heart, kidney and skin transplantation, and can effectively prolong the survival time of the graft. Therefore triptolide has been widely used in inflammatory and autoimmune diseases (Yuan et al., 2019), including rheumatoid arthritis (RA), immune-complex nephritis, systemic lupus erythematosus (SLE), and organ transplantation (Liu, 2011).

It was found that triptolide could bind to PC2 based on extensive chromatographic protein fractionation, MALDI-MS analysis, and Western blot results. As a PC2 agonist, triptolide could restore cytosolic Ca^2+^ release. In *Pkd2* ^+/−^ mouse renal epithelial cells, triptolide increased intracellular Ca^2+^. When the cell line re-expressed *Pkd2*, the intracellular Ca^2+^ level was elevated and apoptosis was increased with triptolide’s incubation (Leuenroth et al., 2007). Therefore, triptolide might inhibit cyst development by activating PC2, thus increasing intracellular Ca^2+^ and inducing caspase pathway. According to some other studies, induction of expression of p53 protein, activation of MAPK pathway and inhibition of NF-κB signaling pathway may also play roles in the progress of apoptosis, which needs to be further explored (Kim et al., 2010; Tai et al., 2010).

Rapamycin, an inhibitor of mTOR, could inhibit the cystic progress of ADPKD kidneys by downregulating cyclin A, cyclin B, cyclin 1D and cyclin E, which are associated with cell cycle, thereby preventing abnormal proliferation of renal epithelial cells (Li A. et al., 2017). Roscovitine, a cycle-dependent kinase inhibitor, has been shown to inhibit the formation of renal cysts in animal models (Billot et al., 2018), further illustrating that cell cycle may be one of targets in inhibiting ADPKD. Considering the cell cycle arrest effect of triptolide in colon cancer, the regulation of cell cycle may be involved in the inhibition of triptolide on the renal cyst formation in animal models. Triptolide arrested cell growth in *Pkd1* ^−/−^ mouse renal epithelial cell line. The up-regulated expression of cyclin p21 was detected (Leuenroth et al., 2007). Furthermore, inhibitory effect of triptolide on cell cycle was also demonstrated in the smooth muscle cells induced by platelet-derived growth factor (PDGF) (He et al., 2020) and renal cell carcinoma cells (Li et al., 2011) through G0/G1 cell cycle arrest, which supports that triptolide may induce cell cycle arrest in ADPKD model.

### Curcumin

Curcumin, a polyphenol diferuloylmethane extracted from the rhizome of *Curcuma longa* plant, has been reported to have multiple effects including anti-oxidation, anti-inflammation and anti-proliferation. Curcumin has shown potential therapeutic effects on various diseases such as neurodegenerative disorders, inflammation-related diseases, fibrosis and cancers *via* regulating NF-κB, Wnt/β-catenin, MAPK and mTOR signaling pathways (Iqbal et al., 2009), which also are involved in the pathogenesis of ADPKD. Previous studies have demonstrated the inhibitory effect of curcumin on cyst growth by inhibiting cell proliferation and promoting epithelial cell differentiation in *Pkd1* deletion mouse models, suggesting its potential to be a natural candidate drug for ADPKD.

In the forskolin-induced MDCK model, which is an *in vitro* cyst model with MDCK cells cultured in 3D collagen gel, the cell proliferation was significantly inhibited by curcumin incubation and the inhibitory effect may be reversible. The Western blot results revealed that in MDCK cells, curcumin could downregulate B-Raf, upregulate Raf-1 and inhibit ERK signalings, indicating that curcumin inhibits cyst development *via* suppressing Ras/B-Raf/MEK/ERK signaling pathway (Gao et al., 2011).

Another crucial modulator of cell proliferation activated in ADPKD is STAT3. An inducible kidney-specific *Pkd1*-deletion mouse model was designed to investigate whether STAT3 signaling was involved in the inhibition of cystogenesis by curcumin. Experimental analysis reflected that activated STAT3 was significantly reduced by curcumin. Therefore, STAT3 inhibition might be a possible mechanism in the curcumin-mediated inhibition on cystogenesis (Leonhard et al., 2011).

MDCK tubule model, in which MDCK cells were incubated in 3T3 conditioned medium, was used to investigate the effect of chemicals on the differentiation of MDCK cells/cysts. Treated with curcumin (at 0.4, 2 or 10 μM) for 12 days, the number of tubule-like structures increased in a dose-dependent manner, and the average length of the longest tubules derived from each MDCK cyst treated by curcumin was longer than those in control group. Consequently, curcumin can promote MDCK cell differentiation, subsequnently inhibiting cyst development (Gao et al., 2011).

### Ginkolide B

Ginkgolide B, a major terpene lactone, is an active component of *Ginkgo biloba*. Ginkgolide B is used as a traditional medicine due to its pharmacological properties such as anti-inflammatory, anti-oxidant, anti-tumor and anti-apoptotic activity (Zhou et al., 2016). Ginkgolide B was also found to inhibit cyst growth in MDCK cyst model and in *Pkd1* knockout mice. Experimental results showed that ginkgolide B inhibited cyst formation and growth in a dose-dependent manner through downregulating Ras/MAPK pathway and the inhibitory effect was reversible (Zhou et al., 2012).

### Stevioside and Its Derivative

Stevioside is a high-sweet, low-calorie sweetener extracted from the leaves and stems of Stevia Rebuadiana plant, which is a herbaceous plant of the composite family. It is degraded by intestinal microflora to its aglycone, steviol, then taken up into the blood circulation. Stevioside has been reported to have properties including anti-hypertension, anti-hyperglycemia, anti-inflammation, anti-tumor, anti-diarrhea and immunity regulation (Chatsudthipong and Muanprasat, 2009). At present, the pharmacological effect and possible mechanisms of stevioside and its derivative steviol in ADPKD have been investigated, and the possible mechanisms in suppressing cyst progression include restraining the cyst fluid secretion and inhibiting tubule cell proliferation.

ADPKD progression contains two key processes, cell proliferation and fluid secretion, involving multiple signaling pathways. The fluid secretion is driven by cAMP-activated transepithelial chloride transport *via* the cystic fibrosis transmembrane conductance regulator (CFTR) chloride channel located at apical membrane of the ADPKD epithelial cells lining the cyst (Li and Sheppard, 2009). CFTR chloride channel has been proposed as a potential target for PKD intervention. Steviol and its derivatives (isosteviol, dihydroisosteviol and 16-oxime isosteviol) inhibited the formation and growth of cysts in the MDCK cyst model, and the inhibitory effect of steviol was the greatest. Furthermore, the underlying mechanisms of the inhibition of steviol on the forskolin-stimulated apical chloride current included direct inhibition of CFTR chloride channel activity and reduction of CFTR expression *via* activating proteasome degradation pathway (Yuajit et al., 2013). In addition to proteasome degradation, an ADPKD mouse model showed that steviol also activated adenosine monophosphate-activated protein kinase (AMPK) signaling pathway, which could subsequently decrease CFTR chloride channel expression. Accordingly, steviol can retard cyst expansion in part by reducing CFTR expression *via* promoting AMPK activity or proteasome-mediated CFTR degradation (Yuajit et al., 2014).

It has been demonstrated that cyst expansion is induced by the excessive secretion of Cl^−^ and water into the cyst lumen through CFTR and aquaporins (AQPs) respectively. Aquaporin 2 (AQP2) is involved in fluid secretion in ADPKD and promotes cyst enlargement. Steviol was found to significantly inhibit the growth of cysts *in vitro* by reducing AQP2 expression in mouse renal cyst epithelial cells. In addition, steviol treatment could increase the expression of enzyme marker of lysosomes, LAMP2, whch also might play a role in AQP2 degradation. Thus, steviol reduced AQP2 expression by reducing AQP2 transcription and promoting proteasome and lysosome-mediated AQP2 degradation, sequentially slowing cyst growth (Noitem et al., 2018).

Stevioside is metabolized into steviol by gut bacteria, which is easily absorbed by the gut and can also reach the kidney (Cosola et al., 2018). In *Pkd1* ^*flox/flox*^: *Pkhd1-Cre* mice, high dose of stevioside could decrease kidney weight and cystic index similar to steviol, but could not improve renal function as steviol. In an orthologous mouse model of human ADPKD (*Pkd1* ^*flox*/*flox*^;*Pkhd1*-*Cre*), stevioside and steviol inhibited the CFTR expression and mTOR/S6K proteins by activating AMP-activated protein kinase, thereby delaying the cyst development through inhibiting proliferation of renal epithelial cells (Yuajit et al., 2014). Moreover, Steviol enhanced the expression of lysosomal enzyme marker LAMP2, indicating the increase of lysosomal degradation of β-catenin, which involves in cell proliferation (Yuajit et al., 2017). In general, stevioside and its derivative, steviol, could regulate pathways of cell proliferation to slow down progression of cysts.

### *G. lucidum* Triterpenoids

*Ganoderma lucidum* is a Chinese traditional medicine. It has been widely used as a dietary supplement or medicine to enhance immunity and therefore improve health for more than 2,000 years in Asia region (Hsu and Yen, 2014). Previous studies have confirmed that *Ganoderma lucidum* consists of a large number of bioactive components, including terpenoids, proteins, polysaccharides, amino acids, flavonoids, alkaloids and steroids (Geng et al., 2020). Pharmacological and clinical studies showed a variety of pharmacological effects of *G. lucidum* including anti-oxidation, anti-inflammation (Zhang et al., 2018), anti-liver disorder (Zhong et al., 2018) and anti-tumor (Chen S. et al., 2017), which were mainly attributed to two major active components: *G. lucidum* polysaccharides and *G. lucidum* triterpenoids (GTs) (Wu et al., 2001; Liang et al., 2019). GTs can also target NF-κB, Ras/MAPK, PI3K/Akt/mTOR and other signalings through G protein-coupled receptor or RTK membrane receptor signal transduction pathways, causing stagnation of cell cycle and inducing tumor cell apoptosis (Gao et al., 2002). Based on the fact that the activated signaling pathways in the pathogenesis of ADPKD are highly similar to that in solid tumor, drugs inhibiting tumor proliferation may have similar effects in the treatment of ADPKD.

To investigate whether GTs have the potential in inhibiting the progression of ADPKD, MDCK cyst model was performed, and GTs were shown to inhibit the formation and growth of cysts (Su et al., 2017). Meanwhile, the embryonic renal cyst model and two rapidly advancing ADPKD mouse models were employed to confirm the inhibitory effect of GTs on renal cysts at organ level and in internal environment. *In vitro* experiments also showed that the GTs could down-regulate the expression of H-ras, B-Raf, *p*-MEK, *p*-ERK, Egr-1 and c-fos, and up-regulate the expression of Raf-1, suggesting that GTs suppressed cell proliferation by down-regulating intracellular excessive accumulation of cAMP and the Ras/MAPK signaling pathway. Meanwhile, GTs promoted epithelial tubule formation in MDCK cells, which also contributes to alleviate the development of cysts by promoting epithelial cell differentiation (Su et al., 2017).

**FIGURE 2:**
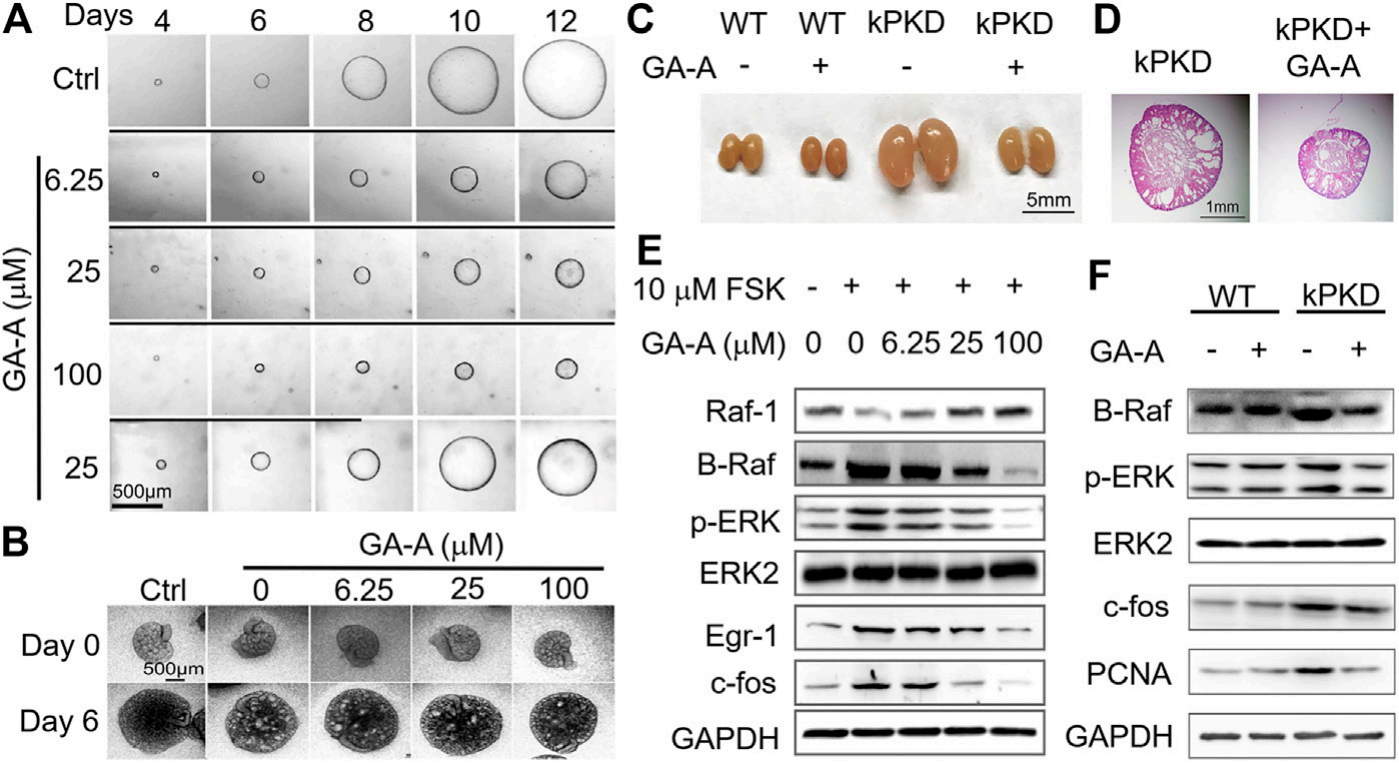
Inhibition of GA-A on the growth of cysts in ADPKD. **(A)** Representative light micrographs of MDCK cysts in collagen gel from 4th to 12th day that were untreated or treated with GA-A at different concentrations. The first row, as the control group, was given only FSK stimulation for 12 days; the second, third, and fourth rows were given FSK and 6.25, 25, 100 μM GA-A co-stimulation from the 5th to 12th day, respectively. In the fifth row, FSK and 25 μM GA-A were co-stimulated from the fifth to eighth day, and the GA-A was washed out from the 9th to 12th day with only FSK. The thick black lines indicate the culture time with GA-A. Scale bar = 500 μm. As the concentration of GA-A increases, the inhibitory effect on cyst growth was more obvious. The cyst growth could be restored after removal of GA-A, indicating that the inhibitory effect of GA-A on cyst growth is reversible. **(B)** Representative graphs of inhibitory effect of different doses of GA-A on mouse embryonic kidney cysts on day 0 and day 6. Embryonic kidneys were cultivated as the negative control (Ctrl) without 100 μM 8-Br-cAMP or exposed to different GA-A concentrations in the presence of 100 μM 8-Br-cAMP. Scale bar = 500 μm. Results showed that GA-A also inhibited the cyst development at the organ level and the inhibitory effect showed a certain dose-effect relationship. **(C)** Mouse kidneys from wild-type (WT) mice and kPKD mice on postnatal day 4 were treated with vehicle or 50 mg/kg/d GA-A for 4 days. Scale bar = 5 mm. Compared with WT mice, the kidney volumes of kPKD mice were significantly increased, and GA-A effectively inhibited the kidney volumes of kPKD mice with no obvious effect on the kidney volume of WT mice. **(D)** Hematoxylin and eosin staining of kidneys in vehicle- or GA-A-treated kPKD mice. Scale bar = 1 mm. The kidney of kPKD mice had obvious cystic structure, and normal tissue structure of the kidney was destroyed. The cyst growth in the kidney of kPKD mice treated with GA-A was effectively slowed. **(E)** Representative Western blotting of signaling proteins in MDCK cells treated with 10 μM FSK and without or with GA-A at different concentrations for 30 min. Results demonstrated that GA-A downregulating the Ras/MAPK signaling pathway in the FSK-treated MDCK cells. **(F)** Representative Western blotting of signaling proteins in mouse kidneys without or with GA-A treatment.

Due to the complexity and structural diversity of triterpene components of *G. lucidum*, screening and identifying the effective monomer components is of great significance for the development of drugs for ADPKD treatment with *G. lucidum* extract. 12 monomer components, including ganoderic acid A, ganoderic acid B, ganoderic acid C2 and ganoderic acid D, were isolated and extracted from GTs. The pharmacodynamic study was carried out in the *in vitro* MDCK cyst model and found that compared with other monomers, ganoderic acid A (GA-A) has a superior inhibitory effect on the development of ADPKD renal cysts, as shown in Figure 2 (Meng et al., 2020). Results showed that GA-A could inhibit the expression of B-Raf, *p*-ERK and c-fos in a dose-dependent manner, with no significant effect on normal cells and the kidney tissue of wild-type mice, indicating that GA-A could inhibit the development of ADPKD cysts by down-regulating the Ras/MAPK signaling pathway. The *in vitro* activities of GA-A and other GT compounds were compared *via* MDCK cyst model and embryonic kidney cyst model, and it was found that the inhibitory effect on cyst of GA-A was better than other compounds (Meng et al., 2020). Therefore, GA-A may be the main effective component in retarding the development of ADPKD cysts, and has the potential to be developed as a therapeutic drug for the treatment of ADPKD.

### Celastrol

Celastrol is another main active ingredient of *Tripterygium wilfordii* in addition to triptolide (Salminen et al., 2010). Previous *in vitro* studies have proved that celastrol has a significant inhibitory effect on the angiogenesis of vascular endothelial cells and the proliferation of endothelial cells. And celastrol showed anti-inflammatory, anti-tumor, anti-obesity properties and the application in autoimmune diseases (Lee et al., 2014; Liu et al., 2015; Shaker et al., 2014; Yu et al., 2018). Celastrol also played a part in regulating autophagy and apoptosis (Liu et al., 2019) and inhibiting cell proliferation in tumor cells (Liu et al., 2019), suggesting that celastrol could possibly possess the therapeutic potential for ADPKD.

Celastrol was identified as a potent inhibitor of cyst growth in both *in vitro* 3D cell model and a *Pkd1*-deficient mouse model (Booij et al., 2019). Considering the anti-inflammatory effect of celastrol in other diseases, the effect of celastrol on pro-inflammatory factors and signaling pathways in ADPKD was investigated. The experimental results indicated that celastrol could suppress the expression of proinflammatory cytokines and macrophage infiltration, and had a tendency to decrease NF-κB with no statistical difference (Chang et al., 2018). According to the knowledge that AMPK activation retards renal cystogenesis *via* inhibiting mTOR-mediated cell proliferation, the level of AMPK phosphorylation was measured with celastrol treatment in the cystic kidneys. The results displayed that celastrol considerably increased AMPK activation. Thus, celastrol exerts a more powerful role in anti-inflammatory effect than in inhibiting cell proliferation. However, 2 mg/kg/d dosage of celastrol may be toxic in female *Pkd1* miR TG mice, and the potential complications should be monitored and paid extra attention to (Chang et al., 2018).

### Saikosaponin-d

Saikosaponin-d, one of the major triterpenoid saponins derived from *Bupleurum falcatum* L (Umbelliferae), has strong pharmacological activities, such as antipyretic, sedative, anti-inflammatory, antibacterial, liver protection, anti-nephritis and immune regulation effects (Cheng et al., 2006; Li C. et al., 2017). Based on previous studies that saikosaponin-d inhibited cell proliferation in three human anaplastic thyroid cancer cell lines and induced G1-phase cell cycle arrest in different cancer cell lines (Liu and Li, 2014), saikosaponin-d can be presumed to be developed as an alternative herbal medicine for ADPKD.

In terms of the pathogenesis of ADPKD, autophagy plays a crucial role in the cystogenesis (Zhu et al., 2017), which is supported by the fact that autophagy activators can slow down the formation and growth of renal cysts, thereby preserving renal function in a *Pkd1* mutant zebrafish model.

In the MDCK cell cyst model, MDCK cells were cultivated for 7 days with forskolin stimulation and formed cysts. With saikosaponin-d treatment, cyst diameters were significantly reduced. The induction of cell cycle arrest of saikosaponin-d was also confirmed in ADPKD cells by accumulation of P27 (Shi et al., 2018). In addition, saikosaponin-d increased autophagy by increasing autophagosome formation and promoted autophagic flux in ADPKD cells. Furthermore, previous experimental results (Wong et al., 2013) showed that saikosaponin-d increased cytosolic Ca^2+^ level and activated autophagy *via* CaMKK β-MPK-mTOR kinase cascade, which may explain the role of saikosaponin-d in the inhibition of cyst growth in ADPKD (Shi et al., 2018).

### Sparganum stoloniferum Buch.-Ham

The Chinese herb *Sparganum stoloniferum* Buch.-Ham (SBH), also called common burred rhizome, consists of several active ingredients including volatile oils and flavonoids, saponins, organic acids, phenylpropanoids and alkaloids (Wang et al., 2015). Recent evidence proved that SBH exerts anticoagulant, antithrombotic, analgesic, anti-inflammatory, antitumor and other pharmacological effects (Sun et al., 2011). The effect of SBH on ADPKD has also drawn the attention.

The direct activation of epithelial growth factor receptor (EGFR) by its ligands is associated with hyperactivated proliferation (Melenhorst et al., 2008), migration, inflammation and fibrosis (Rayego-Mateos et al., 2018) of renal tubular cells. Downregulation of this pathway has been suggested to be involved in the pathogenesis of ADPKD (Torres and Harris, 2006). Based on previous researches, SBH could regulate EGFR activation, therefore explaining the therapeutic potential for ADPKD. In ADPKD cyst-lining epithelial cells, compared with untreated group, SBH could significantly inhibit cell proliferation stimulated by epithelial growth factor (EGF). Moreover, SBH could also repress the phosphorylation of EGFR in cyst-lining epithelial cells stimulated by EGF. Therefore, SBH may inhibit PKD cyst growth by inhibiting phosphorylation of EGFR (Chenggang et al., 2002) and decreasing the proliferation of the cyst-lining epithelial cells.

### Cordyceps sinensis

Cordyceps sinensis (Cordyceps, Dong Chong Xia Cao), a herbal medicine also known as Chinese caterpillar fungus, is one of widely accepted ingredients in traditional Chinese medicine (Zhang et al., 2014). Laboratory and clinical studies have demonstrated that Cordyceps sinensis could improve a wide range of disorders, including respiratory, kidney, liver and cardiovascular diseases and hyperlipidemia (Chen Y. C. et al., 2017). Cordyceps sinensis also showed therapeutic effects on CKD (Deng et al., 2001) by inhibiting mesangial proliferation (Yang et al., 2003) and reducing the accumulation of extracellular matrix in the renal cortex and renal interstitial fibrosis (Dong et al., 2019) with its anti-oxidant (Yamaguchi et al., 2000) and anti-inflammatory properties.

The extract of Cordyceps sinensis, FTY720 (fingolimod), acts as a potent inhibitor of the S1P receptor (S1PR) due to the similar chemical structure to sphingosine-1-phosphate (S1P). In Cy/+ Han:SPRD rat, which is a non-orthologous rat model of ADPKD, FTY720 downregulated the expression of proinflammatory cytokines including IL-6 and TNFα, and attenuated the activation of inflammatory pathways such as the STAT and NF-κB pathways, leading to the inhibition of renal cyst growth and improvement of renal function. As the only clinical drug derived from Cordyceps sinensis, FTY720 may delay the progression in ADPKD through inhibition inflammation *via* reducing the accumulation of S1P (Li et al., 2020).

## Concluding Remarks

Autosomal dominant polycystic kidney disease is a common hereditary kidney disease, which is characterized by progressively enlarged cysts and may eventually develop into end-stage renal failure. Based on the pathogenesis of ADPKD, several drugs are currently under investigation, including vasopressin antagonists, somatostatin analogs and mTOR inhibitors. However, these drugs have some limitations, such as severe liver toxicity and unstable activities. Therefore, searching for effective, stable and safe medicines for ADPKD is imperative. Natural products extracted from herbal medicines, have been drawing extensive attention due to their good efficacy against ADPKD progression and safety in *vivo* studies, such as triptolide, curcumin, ginkolide B, steviol, *G. lucidum* triterpenoids, celastrol, saikosaponin-d, *Sparganum stoloniferum* Buch.-Ham and cordyceps sinensis, as shown in Table 1.

**TABLE 1:** Underlying possible mechanisms of herbal medicine in ADPKD.

Herbal medicines	Major composition	Key mechanism	Effects
Triptolide	diterpene triepoxide	PC2 agonist; Activation of caspase-3	Induction of cell apoptosis;
			Regulation of cell cycle
Curcumin	polyphenol diferuloylmethane	Suppression of Ras/B-Raf/MEK/ERK pathway	Inhibition of cell proliferation;
			Promotion of cell differentiation
Ginkgolide B	terpene lactone	Suppression of Ras/MAPK pathway	Inhibition of cell proliferation
Steviol	aglycone	Inhibition of CFTR;	Restraining of cyst fluid secretion;
		Inhibition of mTOR pathway	Inhibition of cell proliferation;
Ganoderic acid-A	triterpenoid	Suppression of Ras/MAPK pathway	Inhibition of cell proliferation
Celastrol	pentacyclic triterpene	Downregulation of NF-κB	Inhibition of inflammation
Saikosaponin-d	triterpenoid	Inhibition of SERCA	Activation of autophagy
Sparganum stoloniferum Buch.-Ham	burreed rhizome	Inhibition of phosphorylation of EGFR	Inhibition of cell proliferation
Cordyceps sinensis	FTY720 (fingolimod)	Inhibition of S1PR	Inhibition of inflammation

However, the use of herbal medicines still requires careness, since some herbal medicines have a narrow therapeutic window, bringing restrictions to clinical application. Meanwhile, although herbal medicines are realtively safe, they may also exhibit a potential toxic effect at a relatively high dose. For example, the main adverse reactions of triptolide include liver and kidney injuries, and the loss of functions of reproductive system and hematopoietic system. Furthermore, some herbal medicines with complex components lack proper standards to evaluate their pharmacological effects, which limits the clinical use and requires further research to clarify the main ingredients with effectiveness or toxicity.

To sum up, herbal medicines exhibit great therapeutic effects on ADPKD in both *in vivo* and *in vitro* experiments. To develop herbal medicines into effective therapeutic drugs for ADPKD, further studies are required.
